# The Dynamic Analysis of the Pollutant Emissions Impact on Human Health in China Industries Based on the Meta-Frontier DEA

**DOI:** 10.3390/healthcare8010005

**Published:** 2019-12-24

**Authors:** Changfeng Shi, Hui Wu, Yung-Ho Chiu

**Affiliations:** 1 School of Business Administration, Hohai University, Jinling North Road NO.200, Changzhou 213022, China; 20161953@hhu.edu.cn (C.S.); wuh1216@126.com (H.W.); 2 Department of Economics, Soochow University, 56, Kueiyang St., Sec. 1, Taipei 100, Taiwan

**Keywords:** pollutant emissions, two-stage dynamic DEA, meta-frontier, health efficiency, undesirable outputs

## Abstract

Environmental pollutant emissions have become increasingly serious, and the resulting human health problems have become the focus of social attention. In this study, 30 provinces in China were selected as the object of study, SO_2_, NO_X_ (nitrogen oxide), and PM2.5 were taken as undesirable outputs, and a meta-frontier dynamic data envelopment analysis model was adopted to avoid the disadvantages of static analysis. In this paper, energy efficiency, environmental pollution efficiency, and human health efficiency were incorporated into a unified analysis framework by constructing a two-stage model of the production and health stages. The study shows that the total efficiency score of nine provinces and cities, including Beijing, is 1. However, the score of two-stage efficiency in most provinces, such as Anhui, is less than 1, and the score of production efficiency is higher than that of health efficiency. In the second stage, the average efficiency of health expenditure and medical staff input is low, so it is necessary to make targeted improvement. In this regard, it is necessary for the government to increase health expenditure to improve the overall level of health efficiency.

## 1. Introduction

Energy is the material basis for the survival and development of human society and plays an important role in national economic systems. Due to the continuous progress of China’s industrialization and urbanization and the continuous growth of energy demand, the exhaustion of energy resources and environmental pollution have increasingly become the focus of national life and society as a whole. According to the BP (British Petroleum) Statistical Review of World Energy, China’s energy consumption growth rate increased from 3.3% in 2017 to 4.3% in 2018, exceeding the average growth rate of 3.9% of the past decade. Moreover, China is the world’s largest supplier of industrial products, which accounts for about 70% of China’s total energy consumption [1]. This continuous growth of industrial energy consumption has led to increasingly prominent energy environment problems. At present, SO_2_ and NO_X_ emissions and serious haze pollution are affecting people’s health, and industrial pollution emission is one of the main sources of air pollution. A 2013 assessment by the World Health Organization (WHO) International Agency for Research on Cancer concluded that air pollution is carcinogenic to humans. The particulate matter composition of air pollution is closely related to the increased incidence of cancer, especially lung cancer. These threats are likely to undermine the progress made in public health over the past half-century. At present, the analysis of economics, energy, environmental pollution, and human health mainly includes the following three aspects. First, exploring the causal relationship between economic growth, energy consumption, and environmental pollution, such as in Ang [2], Chang [3], and Usman et al. [4]. Second, exploring the energy efficiency of each city or the influencing factors of energy efficiency, such as in Jebali et al. [5], and Yang [6]. Third, Neidell [7], Cao [8], Lou et al. [9], Xue [10], Rajak [11], Gu [12], and Chen [13] studied the relationship between various air pollutants and human health. Due to increasingly prominent haze problems, some scholars have focused on the relationship between PM and human health, such as Cohen [14], Brook [15], Chen [16], and Guan [17].

However, on the one hand, it is known from past research that the evaluation methods of energy efficiency mainly include regression and data envelopment analyses. Among these, efficiency analysis of data envelopment analyses (DEA) is widely used, but most methodologies are mainly static and only discuss the production stage, failing to understand the sustainable development of energy and environment. On the other hand, in recent years, scholars have shifted their attention to health efficiency, but most scholars have not discussed energy consumption, environmental pollution, and human health in connection to health. In order to improve the shortcomings of static analysis in previous studies and further discuss the relationship between environmental pollution and human health, this study is based on the two-stage meta-frontier dynamic DEA model to study the energy, environment, and human health efficiency of 30 provinces in China, and to evaluate the relationship between inter-provincial pollutant emissions and human health efficiency. This study takes into account the impact of environmental pollution caused by energy consumption on human health, and SO_2_, NO_X_, and PM2.5 are considered in environmental pollution. Previous studies have mostly considered the impact of CO_2_ emissions but, although the CO_2_ concentration has increased, it is not sufficiently high to harm human health. Therefore, this paper did not consider the impact of CO_2_ emissions on health efficiency. Due to the lack of relevant statistical data in Tibet, this research excludes Tibet. In addition, this paper makes three contributions. One is to compare the energy, environment, and health efficiency of different provinces spatially, so as to provide more appropriate suggestions for the implementation of policies in each province. Second, this study not only discusses the efficiency of energy and environmental pollution, but also discusses the impact of health expenditure on human health efficiency. Third, the two-stage meta-frontier dynamic DEA model is adopted, which avoids the shortcomings and problems of static analysis. In addition, it improves upon previous defects, namely, that scholars have only considered the evaluation of energy efficiency or the impact of pollution on health and explores the impact of industrial pollution on health efficiency. The study was conducted from 2013 to 2016, with the production stage as the first stage and the health stage as the second stage. Energy, environmental pollution, and health efficiency were evaluated dynamically using a two-stage model. The results showed that the provinces and cities with a total efficiency score of 1 in the four years from 2013 to 2016 include nine provinces and cities, such as Beijing, Guangdong, and Qinghai, mainly developed regions, coastal regions, and northwest China. The efficiency of the second stage is lower than that of the first stage, therefore, low environmental efficiency and health efficiency are the common difficulties faced by most provinces in China. In this regard, it is suggested that low efficiency provinces should optimize and adjust the industrial structure and energy consumption structure, regulate the development of the heavy chemical industry with serious pollution, and further increase the input of total health expenditure, and develop and improve the big health industry.

## 2. Literature Review

According to the past literature, early studies focused on the causal relationship between economic growth, energy consumption, and environmental pollution. For example, Ang [2] studied the dynamic causal relationship between pollutant emissions, energy consumption, and production in France. Chang [3] studied the correlation between China’s carbon dioxide emissions, energy consumption, and economic growth. Usman et al. [4] calculated the correlation degree between energy consumption, environmental pollution, and economic growth through grey correlation analysis. The evaluation of energy efficiency has also gradually emerged. Scholars’ research focuses on the measurement or analysis of influencing factors of energy efficiency in various cities. Among these, Jebali et al. [5] studied the determinants of energy efficiency in Mediterranean countries from 2009 to 2012. Yang [6] applied game crossover efficiency DEA to analyze the total factor energy efficiency of 26 prefecture-level cities in China from 2005 to 2015 under environmental constraints. Armeanu et al. [7] investigated the effects of energy consumption and environmental pollution on economic growth in a sample of 11 states in Central and Eastern Europe from 2000 to 2016, and discussed causality. Haseeb et al. [8] examine the determinants of Research and Development (R and D) expenditure and health expenditure of Association of Southeast Asian Nations (ASEAN) countries.

In recent years, scholars have begun to pay attention to the impact of air pollution on human health from a number of different perspectives. The first assesses the impact of air pollution on human health, such as in Lou et al. [9], who summarize studies on the health effects of temperature and air quality changes directly or indirectly affected by climate change and summarize the limitations of these studies. Xue [10] estimated the relationship between various environmental factors (including air quality) and self-rated mental health scores of more than 20,000 Chinese residents. The second perspective examines the effects of air pollution on mortality or morbidity of various diseases, such as respiratory and cardiopulmonary diseases. Neidell [11] estimated the impact of air pollution on hospitalizations for childhood asthma. Cao [12] examined the relationship between air pollution and mortality, and found a significant correlation between the level of air pollution and mortality from cardiopulmonary diseases and lung cancer. Rajak [13] assessed the impact of short- and long-term exposure to American Associated Press (AAP) on respiratory morbidity, mortality, and premature mortality in exposed populations in India. A third perspective explores the effects of air pollution on the human health of different genders or ages. Gu [14] found that an increase in air pollution concentration significantly reduced the health level of residents. Chen [15] studied the relationship between high environmental air pollution exposure and respiratory health of children with 2532 primary school students, from grades three to five of two schools, with different air pollution levels in Jinan, China, from 2014 to 2016. With the harm of PM2.5 becoming an increasing concern of society, many scholars have also explored the influence of PM on human health. Cohen [16] analyzed fine particle air pollution (PM2.5) and estimated that ambient air pollution caused about 3% of deaths from heart and lung diseases, about 5% of deaths from trachea, bronchus, and lung cancer, and about 1% of deaths from acute tuberculosis. Brook [17] found that decreased PM levels were associated with reduced cardiovascular mortality. Chen [18] found that an increase of the total amount of suspended particles in the air reduced the life expectancy of Chinese residents. Guan [19] assessed the annual health loss and economic impact caused by PM2.5 exposure in Chinese cities from 2015 to 2017. Yang et al. [20] found that the number of cases for PM2.5-related mortality and morbidity during the winter season was about three times as much as that in the summer season in 28 cities.

According to the above research, the relationship between energy consumption and air pollution, and the impact of air pollution on human health, have been widely discussed by scholars. Regarding research methods, since Charnes et al. [21] published the data envelopment analysis (DEA) model, many scholars have proposed improved models. Banker et al. [22] expanded its hypothesis using scale compensation and proposed a Banker, Charnes and Cooper (BCC) model that could measure technical efficiency (TE) and scale efficiency (SE). Network data envelopment analysis (network DEA) was proposed by Färe et al. [23], which remedies the defect that traditional DEA fails to analyze the performance of various departments, but fails to analyze multiple periods. Then, in 2009, Tone and Tsutsui [24] devised the weighted slacks-based measure network data envelopment analysis model. In 2010, Tone and Tsutsui [25] expanded this model into slacks-based dynamic analysis. In 2013, Tone and Tsutsui [26] combined network DEA and dynamic DEA and proposed a weighted slacks-based measure dynamic network DEA data envelopment analysis model. Many applications of the DEA model in energy and health efficiency studies exist, including Zhang [27], who used a dynamic network slacks-based measure (SBM) model to evaluate production and health efficiencies in Chinese cities. Zhou et al. [28] conducted an empirical study on the energy performance of China’s industrial sector from 2010 to 2014 using the index DEA model proposed to convert non-desirable output into desirable output. Djordjevic and Krmac [29] used the non-radial data envelopment analysis (DEA) model to evaluate EEE (i.e., European road, rail, and aviation sectors) at the macro level. Wang et al. [30] measured the static overall efficiency of local government health expenditure (GHE) in each region of China from 2007 to 2016 using data envelopment analysis (DEA). The spatial spillover effect (SSE) of local GHE static total efficiency in each region was measured by building a spatial Dubin model (SDM). Guo et al. [31] used a slacks-based measure (SBM) with undesirable outputs to assess the industrial environmental efficiency of western China during the period 2001–2015. Liu et al. [32] used the combination of a super-slacks-based measure (SBM) model with the Malmquist productivity index (MPI) to evaluate the static health expenditure efficiency (HEE) and dynamic health expenditure efficiency (DHEE) in rural China from 2007 to 2016. Shao et al. [33] evaluated the eco-efficiency of China’s industrial sectors between 2007 and 2015 using the directional distance function (DDF) of network data envelopment analysis (DEA) and a two-stage structure. In addition, Zhang et al. [34], Bigerna et al. [35], and Li et al. also adopted the two-stage DEA model for research. Scholars who have adopted the meta-frontier dynamic DEA model for efficiency studies include Li et al. [36], Zhang et al. [37], Li et al. [38], and Ren et al. [39].

The two-stage meta-frontier dynamic DEA model is adopted in the present study. This model retains the advantages of dynamic continuous analysis, while two-stage analysis will also more comprehensively evaluate the efficiency of energy, the environment, and health.

## 3. Research Methods

### 3.1. Data and Variables

**Figure 1:**
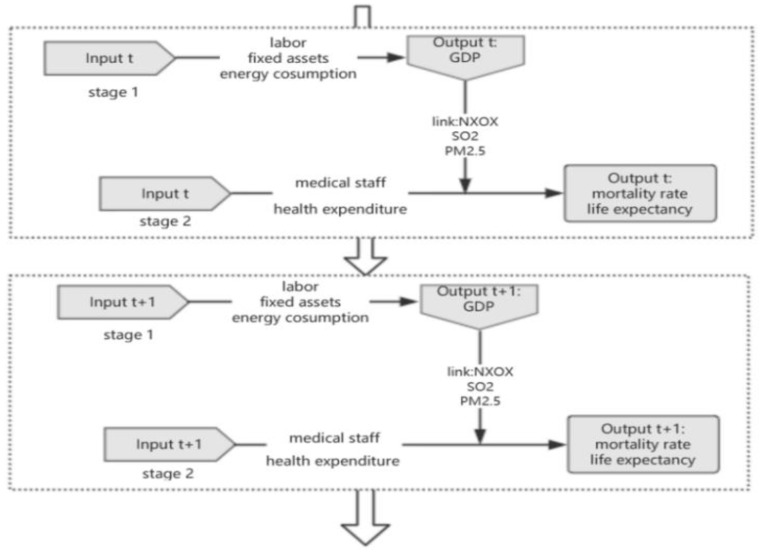
Network model.

Regarding existing national or regional energy efficiency assessment research, Hu and Kao [40] selected labor force, energy consumption, capital, and Gross Domestic Product (GDP) in the first stage, Wang et al. [41] selected labor force, energy consumption, capital, and GDP in the first stage, and Chen and Liu [42] selected energy consumption, labor force, and fixed assets as inputs, and GDP as output. In this paper, industrial labor, energy consumption, industrial fixed assets, and industrial GDP are selected as the inputs and output in the first stage, respectively. Ambient air pollution in urban, suburban, and rural areas is estimated to have caused 4.2 million premature deaths globally in 2016 due to exposure to particulate matter of 2.5 microns or smaller (PM2.5), which increases the incidence and mortality of cardiovascular and respiratory diseases and cancers, according to the WHO. Therefore, SO_2_, NO_X_, and PM2.5 were selected as the undesirable outputs in the first stage. In the second stage, the input and output variables were improved on the basis of Zhang and Chiu [27] for health expenditure, medical staff, mortality rate, and life expectancy. The dynamic relationship of each index is shown in Figure 1.

The construction of the indicator system is shown in Table 1.

Details of the input and output variables are as follows.

The input index of the production stage includes industrial labor, industrial fixed assets, and energy consumption. Industrial labor is the total industrial employment in each province and city, in units of ten thousand. Industrial fixed assets are the stock of industrial fixed assets in each province and city, in units of 100 million yuan. Energy consumption is the amount of standard coal consumed by provinces and cities per year, in units of tons. The output indicator in the production stage is industrial GDP (industrial Gross Domestic Product). Industrial GDP refers to the final result of production activities of all industrial units in a certain period calculated by each province and city according to the market price, in units of 100 million yuan. The undesirable output contains NO_X_ emission, SO_2_ emission, and PM2.5 (Particulate Matter) concentration. The unit of NO_X_ emissions is tons, the unit of SO_2_ emissions is tons, and the unit of PM2.5 concentration is micrograms/cubic meters.

Input indicators in the health stage include health expenditure and medical personnel. Health expenditure is the total social and individual medical expenditure of each province and city per year, in units of 100 million yuan. Medical personnel is the total number of medical personnel in each province and city, in units of ten thousand. The output indicators in the health stage are death rate and life expectancy. The unit of death rate is ‰ and the unit of life expectancy is years.

From 2013 to 2016, industrial labor indicators were drawn from annual city statistical yearbooks, the energy index was derived from the China energy statistical yearbook, nitrogen oxide and sulfur dioxide emissions data were taken from the China statistical yearbook, the average concentrations of PM2.5 data were taken from the provincial ecological environment bulletin, and life expectancy data were taken from the provincial statistical yearbook and the province entries in the Thirteenth Five-Year Plan for health family planning career development special planning.

### 3.2. Modified Undesirable Meta Dynamic Network Model

The production stage is the first stage and the health stage is the second stage. In the first stage, industrial labor, investment in industrial fixed assets, and energy consumption are input items, industrial GDP is the output, and the variables of the link production stage and health stage are SO_2_ emission, NO_X_ emission, and PM2.5 annual average concentration. In the second stage, health expenditure and medical staff are the input items, and mortality and life expectancy are the output items.

This study considered both undesirable outputs and regional differences. Based on Tone and Tsutsui’s [26] dynamic SBM, the meta-frontier model of O’Donnell et al. [43], and the undesirable dynamic network model of Chen et al. [42], we established the modified undesirable meta dynamic network model. The modified undesirable meta dynamic network model is detailed as follows: 

Suppose there are *n* number of DMUs (*j =* 1, …, *n*), with each having *k* divisions (*k* = 1, …, *K*) and T time periods (*t =* 1, …, *T*). Each of the *DMUs* has an input and output at time period *t* and a carryover (link) to the next *t +* 1 time period.

Set *m_k_* and *r_k_* to represent the input and output in each division *K*, with (*k, h*) representing divisions *k* to *h* and *L_hk_* being the *k* and *h* division set. The inputs and outputs, links, and carryover definitions are given in the following.

Inputs and outputs:

$X_{ijk}^{t}\in R_{+}^{}\;(i=1,...,m_{k}^{};\;j=1,...,n;K=1...,K;t=1,...,T)$ refers to input *i* at time period *t* for DMU_j_ division *k*

$y_{rjk}^{t}\in R_{+}^{}\;(r=1,...,r_{k}^{};\;j=1,...,n;K=1...,K;t=1,...,T)$ refers to output *r* in time period *t* for DMU_j_ division *k*. If part of the output is not ideal, it is considered an input for the division.

Links:

$Z_{j(kh)t}^{t}\in R_{+}^{}\;(j=1;...;n;l=1;..;L_{hk}^{};t=1;...;T)$ refers to the period *t* links from DMU_j_ division *k* to division *h*, with *L_hk_* being the number of *k* to *h* links.

Carryovers:

$Z_{jkl}^{\left(t,t+1\right)}\in R_{+}^{}\;(j=1,...,n;l=1,..,L_{k}^{};k=1,...k,t=1,...,T-1)$ refers to the carryover of *t* to the *t* + 1 period from DMU_j_ division *k* to division *h*, with *L_k_* being the number of carryover items in division *k.*

$Linkin_{k}$ is the number of input links for each division *k*, $\;Linkout_{k}$ is the number of output links for each division *k*, $\;ngood_{k}$ indicates the number of desirable carryovers for each division *k*, and $\;nbad_{k}$ indicates the number of undesirable carryovers for each division *k*.

Meta-frontier (MF):

It is assumed that all units (*N*) are composed of *DMUs* in g groups (*N* = *N*1 + *N*2 +….+ *NG*), where *y_rj_* and *x_ij_* indicate the output item *r* (*r* = 1, 2, …, *s*) for item *j* (*j =* 1, 2, …, *N*) and input item *i* (*i =* 1, 2, *…*, *m*) for item *j* (*j* = 1, 2, …, *N*) under the meta-frontier. The meta-frontier *k* of *DMU* efficiency is solved using the following linear program (LP):

Objective function:

Overall efficiency:

(1) $$\theta_{0}^{*}=\min\frac{\sum_{t=1}^{T}W^{t}\left[\sum_{k=1}^{K}W^{k}\left[1-\frac{1}{m_{k}+linkin_{k}+ninput_{k}}(\sum_{g=1}^{G}\sum_{i=1}^{m_{k}}\frac{S_{iok}^{t-}}{x_{iokg}^{t}}+\sum_{g=1}^{G}\sum_{{\left(kl\right)}_{l}=1}^{linkin_{l}}\frac{s_{o{\left(kh\right)}_{l}in}^{t}}{z_{o{\left(kh\right)}_{l}ing}^{t}}+\sum_{g=1}^{G}\sum_{k_{l}}^{ngood_{k}}\frac{s_{ok_{l}input}^{\left(t,t+1\right)}}{z_{ok_{l}input}^{\left(t,t+1\right)}})\right]\right]}{\sum_{t=1}^{T}W^{t}\left[\sum_{k=1}^{K}W^{k}\left[1+\frac{1}{r_{1k}+r_{2k}}(\sum_{g=1}^{G}\sum_{r=1}^{r_{1k}}\frac{s_{rokgood}^{t+}}{y_{rokggood}^{t}}+\;\sum_{g=1}^{G}\sum_{r=1}^{r_{2k}}\frac{s_{rokbad}^{t-}}{y_{rokgbad}^{t}})\right]\right]}$$

Subject to:

$x_{ok}^{t}=X_{kg}^{t}\lambda_{kg}^{t}+s_{ko}^{t-}(\forall k,\forall t,\forall g)$
$y_{okgood}^{t}=Y_{kggood}^{t}\lambda_{kg}^{t}-s_{kogood}^{t+}(\forall k,\forall t,\forall g)$
$y_{okbad}^{t}=Y_{kgbad}^{t}\lambda_{kg}^{t}+s_{kobad}^{t-}(\forall k,\forall t,\forall g)$
$e\lambda_{kg}^{t}=1(\forall k,\forall t,\forall g)$
$\lambda_{kg}^{t}\geq 0,s_{ko}^{t-}\geq 0,s_{kogood}^{t+}\geq 0,s_{kobad}^{t-}\geq 0,(\forall k,\forall t,\forall g)$
$Z_{o(kh)in}^{t}=Z_{(kh)ing}^{t}\lambda_{kg}^{t}+S_{o(kh)in}^{t}\;((kh)in=1,...,linkin_{k})$
$Z_{ok_{l}good}^{\left(t,(t+1)\right)}={\sum_{j=1}^{n}z}_{jk_{l}ginput}^{\left(t,(t+1)\right)}\lambda_{jkg}^{t}-s_{ok_{l}input}^{\left(t,(t+1)\right)}$($k_{l}=1,...,ngood_{k};\forall k;\forall t;\forall g)$
$s_{ok_{l}good}^{\left(t,(t+1)\right)}\geq 0,(\forall k,\forall t,\forall g)$

(a) Period and division efficiencies.

The period and division efficiencies are as follows:

(a1) Period efficiency:

(2) $$\partial_{0}^{*}=\min\frac{\sum_{k=1}^{K}W^{k}\left[1-\frac{1}{m_{k}+linkin_{k}+ninput_{k}}(\sum_{g=1}^{G}\sum_{i=1}^{m_{k}}\frac{S_{iok}^{t-}}{x_{iokg}^{t}}+\sum_{g=1}^{G}\sum_{{\left(kl\right)}_{l}=1}^{linkin_{l}}\frac{s_{o{\left(kh\right)}_{l}in}^{t}}{z_{o{\left(kh\right)}_{l}ing}^{t}}+\sum_{g=1}^{G}\sum_{k_{l}}^{ninput_{k}}\frac{s_{ok_{l}input}^{\left(t,t+1\right)}}{z_{ok_{l}ginput}^{\left(t,t+1\right)}})\right]}{\sum_{k=1}^{K}W^{k}\left[1+\frac{1}{r_{1k}+r_{2k}}(\sum_{g=1}^{G}\sum_{r=1}^{r_{1k}}\frac{s_{rokgood}^{t+}}{y_{rokggood}^{t}}+\;\sum_{g=1}^{G}\sum_{r=1}^{r_{2k}}\frac{s_{rokbad}^{t-}}{y_{rokgbad}^{t}})\right]}$$

(a2) Division efficiency:

(3) $$\varphi_{0}^{*}=\min\frac{\sum_{t=1}^{T}W^{t}\left[1-\frac{1}{m_{k}+linkin_{k}+ninput_{k}}(\sum_{g=1}^{G}\sum_{i=1}^{m_{k}}\frac{S_{iok}^{t-}}{x_{iokg}^{t}}+\sum_{g=1}^{G}\sum_{{\left(kl\right)}_{l}=1}^{linkin_{l}}\frac{s_{o{\left(kh\right)}_{l}in}^{t}}{z_{o{\left(kh\right)}_{l}ing}^{t}}+\sum_{g=1}^{G}\sum_{k_{l}}^{ninput_{k}}\frac{s_{ok_{l}input}^{\left(t,t+1\right)}}{z_{ok_{l}ginput}^{\left(t,t+1\right)}})\right]}{\sum_{t=1}^{T}W^{t}\left[1+\frac{1}{r_{1k}+r_{2k}}(\sum_{g=1}^{G}\sum_{r=1}^{r_{1k}}\frac{s_{rokgood}^{t+}}{y_{rokggood}^{t}}+\;\sum_{g=1}^{G}\sum_{r=1}^{r_{2k}}\frac{s_{rokbad}^{t-}}{y_{rokgbad}^{t}})\right]}$$

(a3) Division period efficiency:

(4) $$\rho_{0}^{*}=\min\frac{\left[1-\frac{1}{m_{k}+linkin_{k}+ninput_{k}}(\sum_{g=1}^{G}\sum_{i=1}^{m_{k}}\frac{S_{iok}^{t-}}{x_{iokg}^{t}}+\sum_{g=1}^{G}\sum_{{\left(kl\right)}_{l}=1}^{linkin_{l}}\frac{s_{o{\left(kh\right)}_{l}in}^{t}}{z_{o{\left(kh\right)}_{l}ing}^{t}}+\sum_{g=1}^{G}\sum_{k_{l}}^{ninput_{k}}\frac{s_{ok_{l}input}^{\left(t,t+1\right)}}{z_{ok_{l}ginput}^{\left(t,t+1\right)}})\right]}{\left[1+\frac{1}{r_{1k}+r_{2k}}(\sum_{g=1}^{G}\sum_{r=1}^{r_{1k}}\frac{s_{rokgood}^{t+}}{y_{rokggood}^{t}}+\;\sum_{g=1}^{G}\sum_{r=1}^{r_{2k}}\frac{s_{rokbad}^{t-}}{y_{rokgbad}^{t}})\right]}$$

From the above, the overall efficiency, period efficiency, division efficiency, and division period efficiency can be obtained using the meta-frontier model.

Group-frontier (GF):

As each DMU under the group frontier chooses the most favorable final weighted output, the DMU efficiencies under the group frontier are solved using the following equations:

(**a**) The objective function.

Overall efficiency:

(5) $$\theta_{0}^{*}=\min\frac{\sum_{t=1}^{T}W^{t}\left[\sum_{k=1}^{K}W^{k}\left[1-\frac{1}{m_{k}+linkin_{k}+ninput_{k}}(\sum_{i=1}^{m_{k}}\frac{S_{iok}^{t-}}{x_{iok}^{t}}+\sum_{{\left(kh\right)}_{l}=1}^{linkin_{k}}\frac{s_{o{\left(kh\right)}_{l}in}^{t}}{z_{o{\left(kh\right)}_{l}in}^{t}}+\sum_{k_{l}}^{ninput_{k}}\frac{s_{ok_{l}input}^{\left(t,t+1\right)}}{z_{ok_{l}input}^{\left(t,t+1\right)}})\right]\right]}{\sum_{t=1}^{T}W^{t}\left[\sum_{k=1}^{K}W^{k}\left[1+\frac{1}{r_{1k}+r_{2k}}(\sum_{r=1}^{r_{1k}}\frac{s_{rokgood}^{t+}}{y_{rokgood}^{t}}+\;\sum_{r=1}^{r_{2k}}\frac{s_{rokbad}^{t-}}{y_{rokbad}^{t}})\right]\right]}$$

$x_{ok}^{t}=X_{k}^{t}\lambda_{k}^{t}+s_{ko}^{t-}(\forall k,\forall t)$
$y_{okgood}^{t}=Y_{kgood}^{t}\lambda_{k}^{t}-s_{kogood}^{t+}(\forall k,\forall t)$
$y_{okbad}^{t}=Y_{kbad}^{t}\lambda_{k}^{t}+s_{kobad}^{t-}(\forall k,\forall t)$
$e\lambda_{k}^{t}=1(\forall k,\forall t)$
$\lambda_{k}^{t}\geq 0,s_{ko}^{t-}\geq 0,s_{kogood}^{t+}\geq 0,s_{kobad}^{t-}\geq 0,(\forall k,\forall t)$
$Z_{o(kh)in}^{t}=Z_{(kh)in}^{t}\lambda_{k}^{t}+S_{o(kh)in}^{t}((kh)in=1,...,linkin_{k})$
$\sum_{j=1}^{n}z_{jk_{1}\alpha }^{\left(t,(t+1)\right)}\lambda_{jk}^{t}=\sum_{j=1}^{n}z_{jk_{1}\alpha }^{\left(t,(t+1)\right)}\lambda_{jk}^{t+1}(\forall k;\forall k_{l};t=1,...,T-1)$
$Z_{ok_{l}good}^{\left(t,(t+1)\right)}={\sum_{j=1}^{n}z}_{jk_{l}good}^{\left(t,(t+1)\right)}\lambda_{jk}^{t}-s_{ok_{l}good}^{\left(t,(t+1)\right)}k_{l}=1,...,ngood_{k};\forall k;\forall t)$
$s_{ok_{l}good}^{\left(t,(t+1)\right)}\geq 0,(\forall k_{l};\forall t)$

(**b**) Period and division efficiencies.

The period and division efficiencies are as follows:

(**b1**) Period efficiency:

(6) $$\partial_{0}^{*}=\min\frac{\sum_{k=1}^{K}W^{k}\left[1-\frac{1}{m_{k}+linkin_{k}+ninput}(\sum_{i=1}^{m_{k}}\frac{S_{iok}^{t-}}{x_{iok}^{t}}+\sum_{{\left(kh\right)}_{l}=1}^{linkin_{k}}\frac{s_{o{\left(kh\right)}_{l}in}^{t}}{z_{o{\left(kh\right)}_{l}in}^{t}}+\sum_{k_{l}}^{ninput_{k}}\frac{s_{ok_{l}input}^{\left(t,t+1\right)}}{z_{ok_{l}input}^{\left(t,t+1\right)}})\right]}{\sum_{k=1}^{K}W^{k}\left[1+\frac{1}{r_{1k}+r_{2k}{}_{k}}(\sum_{r=1}^{r_{1k}}\frac{s_{rokgood}^{t+}}{y_{rokgood}^{t}}+\;\sum_{r=1}^{r_{2k}}\frac{s_{rokbad}^{t-}}{y_{rokbad}^{t}})\right]}$$

(**b2**) Division efficiency:

(7) $$\phi_{0}^{*}=\min\frac{\sum_{t=1}^{T}W^{t}\left[1-\frac{1}{m_{k}+linkin_{k}+ninput_{k}}(\sum_{i=1}^{m_{k}}\frac{S_{iok}^{t-}}{x_{iok}^{t}}+\sum_{{\left(kh\right)}_{l}=1}^{linkin_{k}}\frac{s_{o{\left(kh\right)}_{l}in}^{t}}{z_{o{\left(kh\right)}_{l}in}^{t}}+\sum_{k_{l}}^{ninput_{k}}\frac{s_{ok_{l}input}^{\left(t,t+1\right)}}{z_{ok_{l}input}^{\left(t,t+1\right)}})\right]}{\sum_{t=1}^{T}W^{t}\;\left[1+\frac{1}{r_{1k}+r_{2k}}(\sum_{r=1}^{r_{1k}}\frac{s_{rokgood}^{t+}}{y_{rokgood}^{t}}+\;\sum_{r=1}^{r_{2k}}\frac{s_{rokbad}^{t-}}{y_{rokbad}^{t}})\right]}$$

(**b3**) Division period efficiency:

(8) $$\rho_{0}^{*}=\min\frac{1-\frac{1}{m_{k}+linkin_{k}+ninput_{k}}(\sum_{i=1}^{m_{k}}\frac{S_{iok}^{t-}}{x_{iok}^{t}}+\sum_{{\left(kh\right)}_{l}=1}^{linkin_{k}}\frac{s_{o{\left(kh\right)}_{l}in}^{t}}{z_{o{\left(kh\right)}_{l}in}^{t}}+\sum_{k_{l}}^{ninput_{k}}\frac{s_{ok_{l}input}^{\left(t,t+1\right)}}{z_{ok_{l}input}^{\left(t,t+1\right)}})}{1+\frac{1}{r_{1k}+r_{2k}}(\sum_{r=1}^{r_{1k}}\frac{s_{rokgood}^{t+}}{y_{rokgood}^{t}}+\;\sum_{r=1}^{r_{2k}}\frac{s_{rokbad}^{t-}}{y_{rokbad}^{t}})}$$

From the above results, the overall efficiency, the period efficiency, the division efficiency, and the division period efficiency are obtained.

### 3.3. Industrial Labor, Industrial Fixed Assets, Energy Consumption, Industrial GDP, SO2, NOx, PM2.5, Health Expenditure, Medical Staff, Mortality, and Life Expectancy Efficiency

#### 3.3.1. The Input Efficiency of the First Stage

$$\mathrm{Industrial}\;\mathrm{labor}\;\mathrm{efficiency}\;=\;\frac{\mathrm{Target}\;\mathrm{industrial}\;\mathrm{labor}\;\mathrm{input}\left(i,t\right)}{\mathrm{Actual}\;\mathrm{industrial}\;\mathrm{labor}\;\mathrm{input}\left(i,t\right)}$$

$$\mathrm{Industrial}\;\mathrm{asset}\;\mathrm{efficiency}\;=\;\frac{\mathrm{Target}\;\mathrm{industrial}\;\mathrm{asset}\;\mathrm{input}\left(i,t\right)}{\mathrm{Actual}\;\mathrm{industrial}\;\mathrm{asset}\;\mathrm{input}\left(i,t\right)}$$

$$\mathrm{energy}\;\mathrm{efficiency}\;=\;\frac{\mathrm{Target}\;\mathrm{energy}\;\mathrm{input}\left(i,t\right)}{\mathrm{Actual}\;\mathrm{energy}\;\mathrm{input}\left(i,t\right)}$$

#### 3.3.2. The Output Efficiency of the First Stage

$$\mathrm{Industrial}\;\mathrm{GDP}\;\mathrm{efficiency}\;=\;\frac{\mathrm{Actual}\;\mathrm{industrial}\;\mathrm{GDP}\;\mathrm{output}\left(i,t\right)}{\mathrm{Target}\;\mathrm{industrial}\;\mathrm{DP}\;\mathrm{output}\left(i,t\right)}$$

$${\mathrm{SO}}_{2}\;\mathrm{efficiency}\;=\;\frac{{\mathrm{Target}\;\mathrm{SO}}_{2}\;\mathrm{undesirable}\;\mathrm{output}\left(i,t\right)}{{\mathrm{Actual}\;\mathrm{SO}}_{2}\;\mathrm{undesirable}\;\mathrm{output}\left(i,t\right)}$$

$${\mathrm{NO}}_{X}\;\mathrm{efficiency}\;=\;\frac{{\mathrm{Target}\;\mathrm{NO}}_{X}\;\mathrm{undesirable}\;\mathrm{output}\left(i,t\right)}{{\mathrm{Actual}\;\mathrm{NO}}_{X}\;\mathrm{undesirable}\;\mathrm{output}\left(i,t\right)}$$

#### 3.3.3. The Input Efficiency of the Second Stage

$$\mathrm{Health}\;\mathrm{expenditure}\;\mathrm{efficiency}\;=\;\frac{\mathrm{Target}\;\mathrm{Health}\;\mathrm{expenditure}\;\mathrm{input}\left(i,t\right)\;}{\mathrm{Actual}\;\mathrm{Health}\;\mathrm{expenditure}\;\mathrm{input}\left(i,t\right)}$$

$$\mathrm{Medical}\;\mathrm{staff}\;\mathrm{efficiency}\;=\;\frac{\mathrm{Target}\;\mathrm{Medical}\;\mathrm{staff}\;\mathrm{input}\left(i,t\right)}{\mathrm{Actual}\;\mathrm{Medical}\;\mathrm{staff}\;\mathrm{input}\left(i,t\right)\;}$$

#### 3.3.4. The Output Efficiency of the Second Stage

$$\mathrm{Mortality}\;\mathrm{rate}\;\mathrm{efficiency}\;=\;\frac{\mathrm{Actual}\;\mathrm{Mortality}\;\mathrm{rate}\;\mathrm{output}\left(i,t\right)\;}{\mathrm{Target}\;\mathrm{Mortality}\;\mathrm{rate}\;\mathrm{output}\left(i,t\right)}$$

$$\mathrm{Life}\;\mathrm{expectancy}\;\mathrm{efficiency}\;=\;\frac{\mathrm{Target}\;\mathrm{Life}\;\mathrm{expectancy}\;\mathrm{output}\left(i,t\right)\;}{\mathrm{Actual}\;\mathrm{Life}\;\mathrm{expectancy}\;\mathrm{output}\left(i,t\right)}$$

If the target index is equal to the actual index value, the index efficiency is equal to 1. In the positive index, if the target index value is less than the actual index value, the index efficiency is less than 1, that is, the index efficiency is low. In the inverse index, if the target value is less than the actual index value, the index efficiency is less than 1, that is, the index efficiency is low.

## 4. Empirical Study

### 4.1. Statistical Analysis of Input-Output Indicators

**Figure 2:**
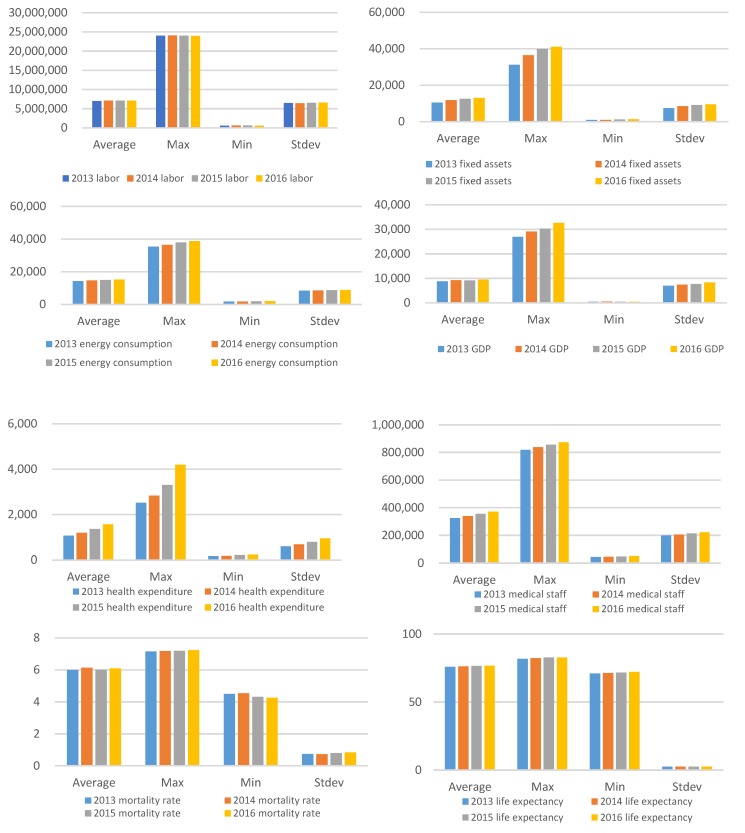
Input-output statistics from 2013 to 2016.

Figure 2 shows statistics on industrial labor, industrial fixed assets, energy consumption, industrial GDP, health expenditure, health care workers, mortality, and life expectancy. According to the statistical analysis results of all indicators, from 2013 to 2016, health expenditure increased the most significantly, and the average and maximum values both significantly increased annually. The maximum value of the stock of industrial fixed assets increased by a large margin, and the annual average also gradually increased. The increase of the stock of industrial fixed assets can promote the long-term growth of productivity to some extent, thus promoting economic growth. However, with the development of the industrial industry, the investment stock of fixed assets also reached a high level. For some areas, whether the increasing stock of fixed assets will bring about a decline in marginal utility remains to be measured.

The maximum annual growth rate of industrial GDP is inferior to the stock of industrial fixed assets, and the maximum growth rate is the most significant, while the average value does not change much, and the gap between the maximum value and the minimum value also increases annually. It can be seen that the economic development speed of different provinces and cities is different, and the growth rate of some provinces and cities is higher than the average level, which leads to the widening of the economic gap.

The average, maximum, and deviation of energy consumption and medical staff from 2013 to 2016 showed a trend of increasing annually, but the increase was not significant. The average death rate fluctuates, with the maximum value increasing annually, but the minimum value decreased, indicating that the development of the economy and medical treatment contributed to the reduction of the death rate. The average, maximum, and minimum of life expectancy increased slightly, and the difference between the maximum and minimum was not obvious. This reflects that the quality of life of the people improved, and the degree of safety also increased, thus improving the life expectancy of the people. However, the resulting aging problem has also received extensive attention from the government and society in recent years.

### 4.2. Total Efficiency Score and Ranking of Cities from 2013 to 2016

Table 2 shows the total efficiency score and ranking of 30 Chinese provinces from 2013 to 2016. The four-year total efficiency score was obtained by geometric weighting, including stage weighting and time weighting. According to the data in the table, Beijing, Guangdong, Hainan, Inner Mongolia, Ningxia, Qinghai, Shanghai, Xinjiang, and Tianjin scored a total efficiency of 1 in the past four years. In addition, the total efficiency of these nine provinces was 1 every year, from 2013 to 2016.

Shaanxi, Gansu, Qinghai, Ningxia, and Xinjiang are in the northwest of China and have the highest reserves of various energy resources in China. To some extent, the utilization efficiency of energy and resources in northwest China affects the local economic development and ecological environment. The total efficiency score of Qinghai, Ningxia, and Xinjiang has reached 1. In 2013–2016, the efficiency score of Shaanxi fluctuated between 0.85–0.94 each year, while the total efficiency score of the four years was only 0.78, which indicates that there was still a large gap in the stage of Shaanxi and there was still a lot of room for improvement. The annual total efficiency score of Gansu was much lower than that of other provinces, and showed a trend of fluctuation and decline, which should be paid attention to. Except for northwest China, the total efficiency scores of Fujian, Jilin, and Jiangsu were better, all above 0.7. Among these, the total efficiency score of Jiangsu province was 0.86, and the total efficiency score of each year was greater than 0.9. The lower ranking provinces were Anhui, Gansu, Guizhou, Hebei, Henan, Heilongjiang, Shanxi, Sichuan, Yunnan, and Chongqing. The total efficiency score of these 10 provinces was less than 0.5 in four years, but the total efficiency of Anhui and Chongqing was more than 0.5 in each year. The large gap between stages led to the low total efficiency score in four years, while the annual efficiency in Gansu, Heilongjiang, and Shanxi was low and showed a downward trend.

From the trend of time series scores, only Guangxi, Guizhou, and Hubei showed a continuous increase in the total efficiency scores, while Anhui, Hebei, Henan, Zhejiang, and Chongqing showed a fluctuating increase. Jilin had a large fluctuation range. Its total efficiency score reached 1 in 2013, but plummeted to 0.78 in 2014, bounced back to 0.96 in 2015, and dropped to 0.8 in 2016, with a fluctuation range of about 0.2.

In general, except for the 13 provinces with high scores, the remaining provinces with scores below 0.7 showed great room for improvement. Among them, the efficiency in Gansu was on a downward trend, falling to 0.37 in 2016, far behind the national average score.

### 4.3. Group Total Efficiency Score from 2013 to 1016

In this paper, 30 provinces are divided into two categories according to the size of industrial output. Among them, the first category comprised provinces with a high industrial output value (group 1): Beijing, Shanxi, Inner Mongolia, Jilin, Heilongjiang, Guangxi, Hainan, Chongqing, Guizhou, Yunnan, Shaanxi, Gansu, Qinghai, Ningxia, and Xinjiang. The other category comprised provinces with a lower industrial output (group 2): Tianjin, Hebei, Liaoning, Shanghai, Jiangsu, Zhejiang, Anhui, Fujian, Jiangxi, Shandong, Henan, Hubei, Hunan, Guangdong, and Sichuan.

Table 3 lists the total efficiency of groups from 2013 to 2016. According to the table, the total efficiency score of group 1 is slightly higher than that of group 2, and the average score of both groups is greater than 0.8. However, the overall efficiency scores of the provinces in group 1 rose slightly between 2013 and 2015, but declined in 2016. The total efficiency score of group 2 was not significantly different from that of group 1 over the past four years. Although the difference between the total efficiency scores of group 1 and group 2 decreased, the total efficiency score of 2016 barely improved compared with that of 2013.

### 4.4. Annual Efficiency Analysis of Each Stage

Table A1 lists the total efficiency score and ranking of each province in the four years, as well as the efficiency score, average efficiency score, and ranking of each province in the first stage and the second stage from 2013 to 2016.

Provinces with an efficiency of 1 in the first stage include Beijing, Fujian, Guangdong, Hainan, Jiangsu, Inner Mongolia, Ningxia, Qinghai, Shaanxi, Shanghai, Tianjin, and Xinjiang. The reason is that Beijing, Tianjin, Shanghai, Guangdong, and other provinces with a good economic foundation have abundant capital and technology accumulation, and the main industries are technology- and capital-intensive, so the industrial energy utilization efficiency is high. Influenced by policies of western development, the industrial upgrade of Shaanxi, Qinghai, and others has relied on resources. The low-level industrial structure has been gradually transformed into a higher-level industrial structure with high industrial energy efficiency. 

In the first stage, the four provinces with the lowest efficiency were Gansu, Shanxi, Yunnan, and Guizhou, all of which were below 0.6, far lower than other provinces. Therefore, these four provinces should further carry out industrial transformation, optimize the energy consumption structure, improve labor productivity, adopt energy-saving technology to improve the coal utilization efficiency, and realize the transformation from extensive economic growth mode to intensive economic growth mode, so as to enhance industrial energy efficiency.

In the second stage, the nine provinces with efficiency scores of 1 were Beijing, Guangdong, Hainan, Inner Mongolia, Ningxia, Qinghai, Shanghai, Tianjin, and Xinjiang. Compared with the first stage, three provinces had an efficiency score of less than 1 in the second stage: Fujian, Jiangsu, and Shaanxi. Among these, the efficiency score of Fujian and Shaanxi in the second stage was below 0.8. This is because the industrial structure of these two provinces is still dominated by secondary industry, most of their economic growth comes from secondary industry, and there is a large bottleneck in industrial transformation. At the same time, PM2.5 concentration and industrial emissions, such as sulfur dioxide, continue to rise, resulting in the efficiency of the second stage being much lower than the first stage. The technology base of the emerging industry is weaker than in the Yangtze River and Pearl River delta provinces and cities.

There was a significant difference in the efficiency of the second stage among all provinces. There were only 15 provinces whose environmental efficiency score was higher than 0.7, and five provinces whose environmental efficiency score was lower than 0.4, which was significantly lower than the efficiency of the first stage. It can be seen that low environmental efficiency is a common difficulty faced by most provinces in China. This is because China’s health expenditure on environmental governance, though increasing, has been lower than that of developed countries as a proportion of GDP. Secondly, local governments do not have strong supervision on the emission of pollutants from industrial sectors and fail to resist environmental pollution from the source. Therefore, various supervision mechanisms and legal systems still need to be built and improved. Finally, local governments should also strengthen environmental protection publicity for enterprises and enhance people’s awareness of environmental protection.

### 4.5. Group Annual Efficiency Analysis of Each Stage

Table 4 shows the efficiency of the first stage and the second stage in the provinces with a high industrial output value (group 1) and the provinces with a low industrial output value (group 2) during 2013–2016. From the perspective of the groups, the efficiency score of group 1 in the first stage was not significantly different from that in the second stage, while the efficiency score of group 2 in the second stage was significantly lower than that in the first stage, indicating that there was a lot of room for improvement in the efficiency of group 2 in the second stage. The efficiency score of group 1 in the first and second stages was relatively stable during the four years, with only a small fluctuation. Moreover, the efficiency score in the second stage showed the same trend as that in the first stage, both of which rose slightly in 2014 and then declined. The group 2 efficiency score of the first and second stages showed a general trend of decline. In 2016, the efficiency score of the first and second stages both dropped to the lowest level in four years, and the efficiency score of the first stage dropped to less than 0.8.

In general, the efficiency scores of the two groups in the second stage were lower than the efficiency scores of the first stage, while the gap between the two efficiency scores of group 2 was relatively large. Although the gap narrowed in 2016, it was not caused by the improvement of the efficiency of the second stage, but the decline of the efficiency of the first stage. Therefore, the provinces in group 2 should focus on improving the efficiency score of the second stage while maintaining the efficiency of the first stage. Group 1: although the efficiency of the two stages is better, there is still room for improvement. In recent years, the efficiency has only been maintained, but no improvement has been achieved.

### 4.6. Efficiency Analysis of Input and Output Indicators in the First and Second Stages from 2013 to 2016

#### 4.6.1. Efficiency Score of Input-Output Index in the First Stage

**Figure A1:**
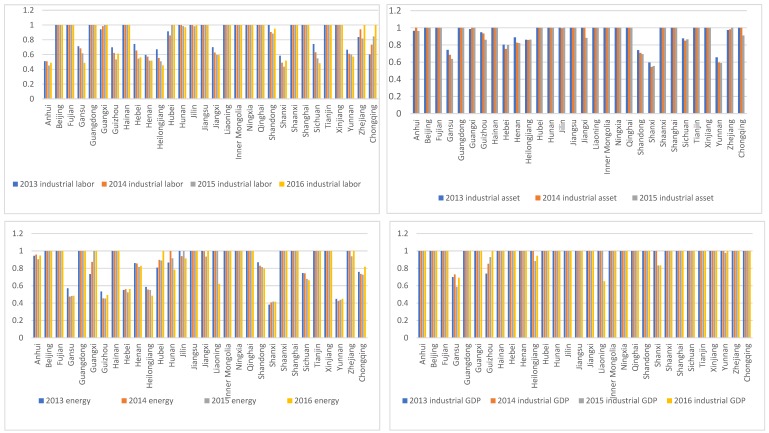
Efficiency of the second stage input-output indicator in 2013–2016.

Figure A1 shows the efficiency score and trend change of industrial labor, energy consumption, and industrial GDP from 2013 to 2016, as well as the efficiency score and trend change of industrial fixed asset input from 2013 to 2015. Among the four indicators, the efficiency score of industrial GDP is the best. The efficiency score of most provinces reached 1 in four years, while the efficiency score of Gansu, Guizhou, Heilongjiang, Liaoning, and Shanxi did not reach 1. Among these, the efficiency score of industrial GDP in Gansu is far lower than that of other provinces. On the one hand, this is due to the fact that the regions with relatively backward economic development in Gansu, such as Longnan and Dingxi, are located in remote areas and have long been dominated by small-scale peasant or natural economies, with a relatively weak economic foundation and a low industrialization level. On the other hand, the resource allocation efficiency of Gansu is low and the industrial structure transformation is sluggish, which results in industrial GDP having far lower efficiency than the average level. This shows that the development of Gansu’s secondary industry is not mature, but also reflects the unreasonable industrial structure. In contrast, Heilongjiang, Liaoning, and Shanxi saw their industrial GDP efficiency reach 1 in four years, but decline in 2016.

From 2013 to 2015, the efficiency of industrial fixed assets was relatively good, and the annual efficiency score of most provinces reached 1. Only Gansu, Hebei, Shandong, Shanxi, and Yunnan scored under 0.8 in efficiency of industrial fixed assets.

Twelve provinces scored 1 in energy efficiency over a four-year period. The six provinces with the lowest energy efficiency scores were Gansu, Guizhou, Hebei, Heilongjiang, Shanxi, and Yunnan. As a major coal producing province, Shanxi has the worst energy utilization efficiency. Due to its abundant coal resources, its energy utilization is extensive, which leads to energy waste and environmental pollution. Shanxi should focus on strengthening environmental governance, reducing the emission of pollutants in the process of energy consumption, and strengthening the supervision of pollutant treatment to improve energy and environmental efficiency.

The efficiency score of the industrial labor force varies from province to province, and the difference between different provinces is larger than the other three first-stage indicators. The efficiency of the industrial labor force in most provinces with an efficiency of less than 1 is around 0.7. Therefore, compared with the efficiency of industrial fixed asset investment and industrial GDP, the efficiency of the industrial labor force has a lot of room for improvement.

#### 4.6.2. Efficiency Score of Input-Output Index in the Second Stage

**Figure A2:**
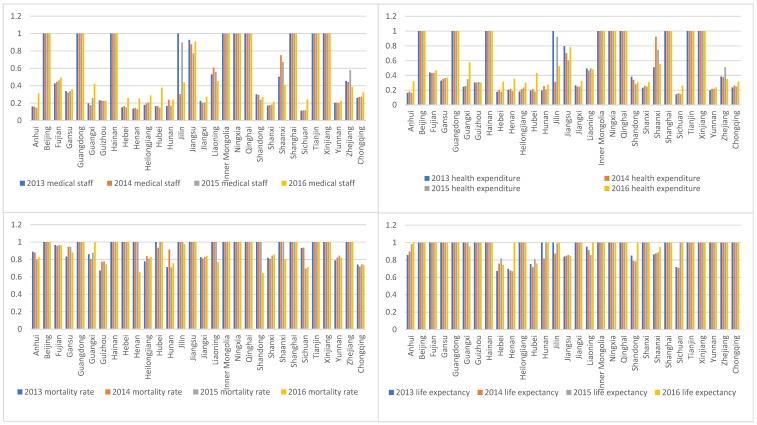
Efficiency of the second stage input-output indicator in 2013–2016.

Figure A2 shows the health expenditure efficiency, medical staff efficiency, mortality efficiency, and life expectancy efficiency scores from 2013 to 2016. From the chart, it can be seen intuitively that the average efficiency level of the input index in the second stage is relatively low. The input efficiency of most provinces in the second stage is less than 0.4, while the output efficiency score is good, both of which reach 0.6 to 1, indicating a huge imbalance.

In most provinces, health expenditure and medical staff input increased, but their efficiency scores were not effectively improved. Excluding Beijing, Guangdong, Hainan, and other provinces where the efficiency is 1, the second-stage investment efficiency of other provinces is far lower than the first-stage investment efficiency. Among them, 12 provinces, including Anhui, Gansu, Guizhou, and Hebei, had a health expenditure efficiency of less than 0.4 from 2013 to 2016, while 14 provinces had a medical staff input efficiency of less than 0.4 in the four-year period. This is because China’s share of GDP spent on health is much lower than the global average. Compared with developed countries, China’s big health industry is still in the stage of development, and the overall scale is small. According to the data of the World Bank, in 2016, the proportion of health expenditure to GDP of the United States was 17.07%, ranking first in the world. However, the proportion of health expenditure to GDP of China in 2016 was only 4.98%. Therefore, China has huge room for future growth and development, and optimization of the structure of the medical service market. However, it will be difficult to increase subsidies to effectively reduce the burden of personal hygiene and only the market main body can utilize limited medical resources to provide high-quality medical services with high efficiency, thus improving the efficiency of health expenditure and medical staff efficiency.

#### 4.6.3. Undesirable Output Indicator Efficiency Score

Table A2 shows the efficiency scores of SO_2_ emission, NO_X_ emission, and PM2.5 concentration of each province from 2013 to 2016. Among the three, the PM2.5 concentration efficiency score was better, and the efficiency score was stable in the four-year period, with a PM2.5 efficiency score above 0.9 in each year. From 2013 to 2016, the overall efficiency of NO_X_ emission was inferior to the annual PM2.5 efficiency, but the efficiency score steadily decreased.

From 2013 to 2016, the provinces where SO_2_ had an emission efficiency score of 1 are Beijing, Fujian, Guangdong, Hainan, Jiangsu, Inner Mongolia, Ningxia, Qinghai, Shaanxi, Shanghai, Tianjin, and Xinjiang. In addition to the 12 provinces with a NO_X_ efficiency score of 1 for the four years, there was also Zhejiang. In addition to the 12 provinces with a PM2.5 efficiency score of 1, there were also Liaoning and Zhejiang. In Yunnan, Guizhou, Hebei, Shanxi, and Gansu, SO_2_ emissions, NOx emissions, and efficiency score were far below the average PM2.5 concentration. These provinces should pay attention to the control of industrial emissions of pollutants, while further adjusting the industrial structure, using cleaner energy, and optimizing SO_2_ and NOx emissions and the concentration of the PM2.5 efficiency score.

**Figure A3:**
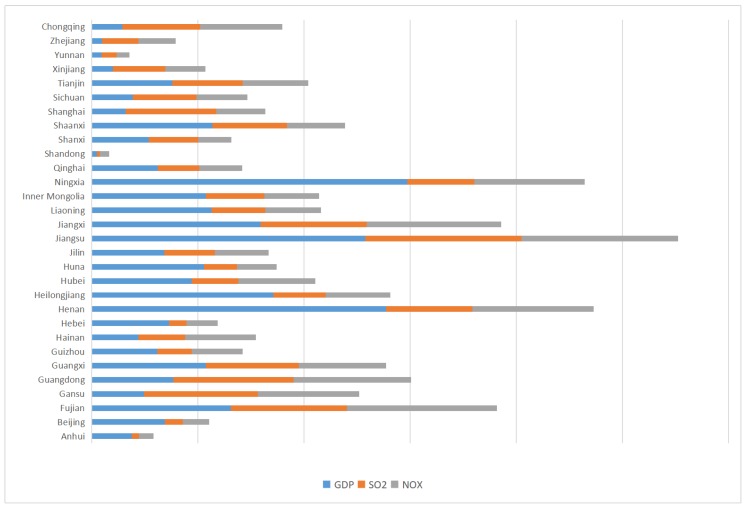
Comparison of the average output values of the output indicators over four years.

Table A3 shows the average industrial GDP, average SO_2_ emissions, average NOx emissions, and average PM2.5 concentration during the four years from 2013 to 2016. Figure A3 shows the relationship between average industrial GDP, average SO_2_ emissions, and average NOx emissions in the past four years. Due to the good results achieved in PM2.5 pollution prevention and control, the efficiency of PM2.5 concentration in most provinces reached 1, so PM2.5 concentration was not taken into consideration. Data and charts show that there is a certain correlation between industrial GDP and pollutant emissions. Higher industrial GDP is usually accompanied by higher pollutant emissions. For example, Fujian, Guangdong, Guangxi, Henan, Jiangsu, and other provinces have higher industrial GDP and correspondingly higher pollutant emissions.

#### 4.6.4. Change Rate of Index Efficiency Ranking after Grouping

Table 5 shows the difference between the four-year average efficiency scores of industrial energy, SO_2_, NO_X_, PM2.5, health expenditure, and medical staff in the provinces with a high industrial output value (group 1) and those with a low industrial output value (group 2) from 2013 to 2016. The positive difference means that the efficiency ranking of the provinces in the group is lower than that outside the group. The selection of these six indicators is due to the high importance of industrial energy efficiency. The efficiency of SO_2_, NO_X_, and PM2.5 represents the environmental pollution treatment efficiency caused by industrial pollution emissions in various provinces, while the score of health expenditure and the efficiency of medical personnel in all efficiency indicators is far lower than that of other indicators. In this calculation, the provinces with an annual index efficiency of 1 were excluded, namely, Beijing, Guangdong, Hainan, Inner Mongolia, Ningxia, Qinghai, Shanghai, Tianjin, and Xinjiang.

From the calculation results, the industrial energy efficiency scores ranked in the group before and after the grouping change were the big provinces: Jiangxi, Jilin, Liaoning, Guangxi, and Fujian. These five provinces improved considerably. Notable is the great progress of the Jiangxi provincial energy efficiency score within the group.

The provinces where SO_2_ efficiency dropped significantly after grouping included Anhui, Fujian, Henan, Hubei, and Shaanxi. Among the provinces with lower industrial output value, Shaanxi dropped 77% compared to prior to grouping, while the provinces with a higher industrial output value after grouping included Jiangxi, Heilongjiang, and Anhui.

The province with a big drop in NOx efficiency after grouping was Zhejiang, while the provinces with a big rise in NOx efficiency after grouping were Liaoning, Guangxi, Jilin, and Fujian.

After grouping, the PM2.5 efficiency scores of all the provinces decreased by less than 15%, while the rank of Anhui, Hubei, Jiangxi, Sichuan, Guangxi, Heilongjiang, Jilin, and Fujian significantly increased.

The provinces that saw a big drop in health expenditure efficiency were Hebei, Shandong, Sichuan, Guangxi, and Anhui, while the provinces that saw a big rise in health expenditure efficiency were Liaoning, Zhejiang, and Jilin. The province with a big drop in efficiency of medical staff after grouping was Guizhou, while the provinces with a big rise in efficiency after grouping were Fujian, Liaoning, and Jilin.

## 5. Conclusions

In this paper, the two-stage meta-frontier dynamic DEA model was adopted to discuss the efficiency of economic, energy, and environmental efficiency and human health in 30 provinces in China and to evaluate the relationship between provincial pollutant emissions and human health efficiency. The following conclusions can be drawn from the analysis:

(1) The provinces with a total efficiency score of 1 in the four years from 2013 to 2016 include nine provinces, such as Beijing, Guangdong, Hainan, Inner Mongolia, Ningxia, Qinghai, Shanghai, Tianjin, and Xinjiang, mainly developed regions, coastal regions, and northwest China. Only Jiangsu and Jilin scored higher than 0.8, and 10 provinces scored lower than 0.5, including Anhui, Gansu, Guizhou, Hebei, Henan, Heilongjiang, Shanxi, Sichuan, Yunnan, and Chongqing. It can be seen that there was still a lot of room for improvement in the total efficiency scores over the four years, among which, Gansu, Heilongjiang, Hunan, Liaoning, and Shandong showed a fluctuating and decreasing trend in the total efficiency scores, which should be paid attention to. It has indicated that the total efficiency scores were not optimistic.

(2) The group’s total efficiency score from 2013 to 2016 was relatively stable during this period, with ups and downs and small fluctuations in both groups. In general, the group with a high industrial output value had higher efficiency score than the group with a low industrial output value, but the efficiency score of the two groups did not make continuous progress in four years.

(3) A total of 12 provinces, including Beijing, Guangdong, and Qinghai, scored 1 in the first stage of efficiency. Each of these provinces has a good economic foundation and relies on resources to realize industrial upgrading. Only nine provinces, including Beijing, Guangdong, and Hainan, scored 1 in the second stage. Due to the continuous rise of industrial exhaust emissions and the continuous increase of PM2.5 concentration, resulting in the efficiency score of the second stage in other provinces being significantly different, and the efficiency of the second stage is lower than that of the first stage. Therefore, low environmental efficiency and health efficiency are the common difficulties faced by most provinces in China.

(4) The efficiency of the second stage is lower than that of the first stage in both groups, and the efficiency of the first stage is not different from that of the second stage in the provinces with a higher industrial output. The efficiency of the second stage in the provinces with a lower industrial output was significantly lower than that in the first stage, so there is a large space for improvement in the efficiency of the second stage in the provinces with a lower industrial output. Although two-stage efficiency was better in provinces with a higher industrial output, there is still room for improvement.

(5) In terms of efficiency of all indicators, the efficiency scores of industrial GDP and industrial fixed assets are relatively good, while the efficiency scores of industrial labor, industrial energy consumption, SO_2_ emission, NO_X_ emission, health expenditure, and medical staff input are relatively low and vary from province to province. Among these, the efficiency of industrial GDP in most provinces reached 1 in the four-year period, and only Gansu, Guizhou, Heilongjiang, Liaoning, and Shanxi failed to reach 1. The health expenditure efficiency score and medical staff input efficiency score were far lower than the other indicators, with the exception of Beijing, Guangdong, Hainan, and other provinces with a health efficiency score of 1, the health expenditure and medical personnel input of other provinces increased year by year, but the efficiency score was not effectively improved. For example, 12 provinces, such as Anhui, Gansu, Guizhou, and Hebei, had a health expenditure efficiency score of less than 0.4 from 2013 to 2016, while 14 provinces had a medical staff efficiency score of less than 0.4 during the four years. From the perspective of two-stage efficiency, the average efficiency level of the input index in the second stage was relatively low. In most provinces, the input efficiency in the second stage was less than 0.4, while the output efficiency score was good, both of which reached 0.6 to 1, which shows the huge imbalance between the two stages.

(6) In Gansu, Guizhou, Yunnan, Shanxi, and Shandong, SO_2_ emissions and NO_X_ emissions efficiency scores were lower than 0.5. In addition to the efficiency score of 1, provinces such as Beijing, Fujian, and the rest of the provinces of SO_2_ emissions, the NO_X_ emissions efficiency score was generally less than 0.8, and for concentrations of PM2.5 efficiency, with the exception of nine provinces, such as Anhui and Gansu, which were below 1, the rest of the provinces scored 1, which showed that China was effective on PM2.5 concentration control. In 2017, China proposed three tough battles. One of the key points of pollution prevention and control is to reduce the concentration of PM2.5 and PM10. According to the research results, the efficiency score of PM2.5 concentration in Hebei, Shandong, Sichuan, and other provinces increased to 1 year by year, which proves that pollution prevention and control is effective.

(7) Although the two-stage meta-frontier dynamic DEA model overcame the shortcomings of static analysis, took into account the influence of energy utilizations, environmental pollution, and human health, and measured the efficiency of the two groups, it also had certain limitations. First, the study only measured the relative efficiency and could not put forward optimization countermeasures for the provinces with an efficiency score of 1. Second, the process was only decomposed in two stages, and the detailed influencing mechanism or path of energy utilization and human health was not involved. Third, since the impact of CO_2_ emissions on human health was indirect, this paper did not consider the impact of CO_2_ emissions on health efficiency, which occupied a major position in environmental pollution. Therefore, it was of certain significance to study the impact of CO_2_ emissions on health efficiency.

### Recommendations

Firstly, Environmental pollution caused by energy consumption will eventually affect human health. Gansu, Shanxi, Yunnan, Guizhou, and Hebei, which scored much lower than other provinces in the first stage, should be further improved by optimizing and adjusting the industrial structure and energy consumption structure of each province. Gansu, Guizhou, Shandong, Shanxi, and Yunnan should regulate the development of the heavy chemical industry with serious pollution, formulate strict industrial access thresholds for areas with sub-standard environmental air quality, strongly support the development of the tertiary industry dominated by the service industry, and gradually develop the low-carbon economy. In addition, local governments should set up corresponding environmental performance evaluation systems, conduct supervision and assessment, and control the emission of pollutants from the source.

Secondly, with the exception of Beijing and Guangdong, which have a total efficiency score of 1, other provinces have to control the total amount and intensity of energy consumption, while accelerating the development of new energy and building a clean, low-carbon, safe, and efficient energy system. In addition, a complete talent training system should be established step-by-step to enhance the accumulation of human capital, so as to accelerate technological innovation and the research and development of clean energy, and reduce the emission of pollutants from energy utilization.

Thirdly, Anhui, Fujian, Gansu, Guangxi, Guizhou, Hebei, Henan, Heilongjiang, Hubei, Hunan, Jiangxi, Liaoning, Shandong, Shanxi, Sichuan, Yunnan, Zhejiang, and Chongqing scored low in the efficiency of health expenditure and input of medical staff. In order to meet the increasing health needs of residents, the input of total health expenses of these provinces should be further increased, and the health industry should be developed and improved. A medical community should be formed through the combination of hospitals with different levels and characteristics to optimize the allocation of medical resources and promote the sharing of resources among different medical institutions. A final goal should be to further balance the layout of medical institutions and narrow the gap between the capabilities of medical institutions at all levels.

## Figures and Tables

**Table 1:** Input and output variables.

	Input Variables	Output Variables	Link	Carry Over
Stage 1	Industrial Labor	Industrial GDP	NO_X_, SO_2_	Industrial Fixed Asset
	Energy Consumption		PM2.5	
Stage 2	Health Expenditure	Mortality Rate		
	Medical Staff	Life Expectancy		

**Table 2:** Efficiency by city from 2013 to 2016.

DMU	Total	Rank	2013	2014	2015	2016
Anhui	1	1	1	1	1	1
Beijing	0.6063	16	0.6014	0.7243	0.8394	0.8906
Fujian	0.3579	28	0.4095	0.4605	0.4756	0.4925
Gansu	1	1	1	1	1	1
Guangdong	0.3619	25	0.4783	0.5068	0.4787	0.5329
Guangxi	0.4046	23	0.5572	0.5827	0.5469	0.6194
Guizhou	0.3597	26	0.5616	0.5454	0.5028	0.4999
Hainan	0.5328	19	0.7039	0.7248	0.7499	0.8288
Hebei	0.5409	18	0.7036	0.8025	0.7504	0.6771
Henan	0.8178	11	1	0.7795	0.9630	0.7988
Heilongjiang	0.8594	10	0.9309	0.9200	0.9043	0.9299
Hubei	0.5060	20	0.6384	0.6498	0.6259	0.6513
Hunan	0.6645	15	0.8926	0.8889	0.8735	0.5685
Jilin	1	1	1	1	1	1
Jiangsu	1	1	1	1	1	1
Jiangxi	1	1	1	1	1	1
Liaoning	0.5535	17	0.7587	0.6685	0.6486	0.6223
Inner Mongolia	0.3114	30	0.4336	0.4342	0.3731	0.3948
Ningxia	0.7836	12	0.8722	0.9364	0.9155	0.8518
Qinghai	1	1	1	1	1	1
Shandong	0.3725	24	0.6051	0.6006	0.5913	0.5996
Shanxi	1	1	1	1	1	1
Shaanxi	1	1	1	1	1	1
Shanghai	0.3529	29	0.4635	0.4624	0.4459	0.4428
Sichuan	0.6885	14	0.8404	0.8661	0.8516	0.8737
Tianjin	0.4930	21	0.5856	0.6375	0.6290	0.6842
Xinjiang	1	1	1	1	1	1
Yunnan	0.6063	16	0.6014	0.7243	0.8394	0.8906
Zhejiang	0.3579	28	0.4095	0.4605	0.4756	0.4925
Chongqing	1	1	1	1	1	1

**Table 3:** 2013–2016 group efficiency.

	Total	2013	2014	2015	2016
Group 1	0.846786	0.849984	0.852496	0.855553	0.829112
Group 2	0.815461	0.808727	0.826673	0.818157	0.808287

**Table 4:** Group efficiency in 2013–2016.

	Stage 1				Stage 2			
	2013	2014	2015	2016	2013	2014	2015	2016
Group 1	0.8762	0.8970	0.8899	0.8878	0.8237	0.8080	0.8212	0.7704
Group 2	0.8366	0.8629	0.8500	0.8488	0.7808	0.7904	0.7864	0.7678

**Table 5:** Rate of change in index efficiency after grouping.

DMU	Cluster	Energy	SO_2_	NO_X_	PM2.5	Health Expenditure	Medical Staff
Anhui	1	13%	40%	13%	−60%	−27%	−13%
Fujian	1	3%	43%	3%	3%	−13%	−43%
Hebei	1	17%	−7%	10%	7%	43%	−7%
Henan	1	10%	33%	17%	10%	−3%	3%
Hubei	1	10%	40%	13%	−53%	−23%	−10%
Hunan	1	−7%	−23%	−3%	7%	−27%	−23%
Jiangsu	1	3%	10%	3%	3%	30%	7%
Jiangxi	1	−40%	−57%	−13%	−77%	−17%	−23%
Liaoning	1	−50%	−23%	−47%	3%	−63%	−37%
Shandong	1	13%	−20%	10%	10%	47%	20%
Sichuan	1	13%	−3%	23%	−57%	50%	−7%
Zhejiang	1	10%	−23%	43%	3%	−33%	7%
Gansu	2	−17%	−27%	−13%	3%	20%	20%
Guangxi	2	−53%	7%	−57%	−83%	40%	17%
Guizhou	2	0%	−7%	13%	13%	17%	33%
Heilongjiang	2	0%	−37%	3%	−50%	7%	20%
Jilin	2	−43%	−17%	−60%	−47%	−57%	−30%
Shanxi	2	0%	−10%	−3%	13%	−10%	−3%
Shaanxi	2	3%	77%	3%	3%	−7%	20%
Anhui	2	−17%	−43%	−27%	3%	40%	−13%
Fujian	2	−70%	−27%	−53%	−93%	−13%	−3%

## Appendix A

**Table A1:** Two stage efficiencies by provinces from 2013 to 2016.

DMU	Total	Rank	Stage 1						Stage 2					
			2013	2014	2015	2016	Average	Rank	2013	2014	2015	2016	Average	Rank
Anhui	0.4325	22	0.7263	0.8102	0.7851	0.800	0.7804	22	0.5017	0.4882	0.4523	0.5770	0.5048	20
Beijing	1	1	1	1	1	1	1	1	1	1	1	1	1	1
Fujian	0.7182	13	1	1	1	1	1	1	0.7603	0.7591	0.7666	0.7768	0.7657	13
Gansu	0.3585	27	0.4484	0.4621	0.3491	0.3700	0.4074	30	0.4028	0.3580	0.3533	0.3611	0.3688	27
Guangdong	1	1	1	1	1	1	1	1	1	1	1	1	1	1
Guangxi	0.6063	16	0.8375	0.9479	1	1	0.9464	16	0.3652	0.5007	0.6788	0.7813	0.5815	16
Guizhou	0.3579	28	0.4549	0.5743	0.5940	0.6550	0.5696	27	0.3641	0.3467	0.3572	0.3301	0.3495	30
Hainan	1	1	1	1	1	1	1	1	1	1	1	1	1	1
Hebei	0.3619	25	0.6458	0.6721	0.6061	0.6408	0.6412	25	0.3109	0.3415	0.3513	0.4251	0.3572	29
Henan	0.4046	23	0.7274	0.7714	0.7189	0.7220	0.7349	23	0.3871	0.3941	0.3748	0.5167	0.4182	25
Heilongjiang	0.3597	26	0.6278	0.6563	0.5636	0.5670	0.6037	26	0.4954	0.4345	0.4419	0.4328	0.4512	22
Hubei	0.5328	19	0.8607	0.9184	0.9629	1	0.9355	17	0.5471	0.5312	0.5369	0.6576	0.5682	17
Huna	0.5409	18	0.9327	1	0.9663	0.9177	0.9542	14	0.4744	0.6050	0.5345	0.4366	0.5126	18
Jilin	0.8178	11	1	0.9797	0.9919	0.9710	0.9857	13	1	0.5793	0.9341	0.6265	0.7850	12
Jiangsu	0.8594	10	1	1	1	1	1	1	0.8618	0.8401	0.8086	0.8598	0.8426	10
Jiangxi	0.5060	20	0.8498	0.8746	0.8431	0.8257	0.8483	19	0.4271	0.4250	0.4087	0.4769	0.4344	24
Liaoning	0.6645	15	1	1	1	0.5693	0.8923	18	0.7851	0.7778	0.7470	0.5677	0.7194	15
Inner Mongolia	1	1	1	1	1	1	1	1	1	1	1	1	1	1
Ningxia	1	1	1	1	1	1	1	1	1	1	1	1	1	1
Qinghai	1	1	1	1	1	1	1	1	1	1	1	1	1	1
Shandong	0.5535	17	0.9347	0.8242	0.8009	0.8132	0.8433	20	0.5827	0.5129	0.4963	0.4315	0.5059	19
Shanxi	0.3114	30	0.4832	0.4972	0.3874	0.4131	0.4453	29	0.3839	0.3711	0.3588	0.3764	0.3726	26
Shaanxi	0.7836	12	1	1	1	1	1	1	0.7443	0.8727	0.8311	0.7035	0.7879	11
Shanghai	1	1	1	1	1	1	1	1	1	1	1	1	1	1
Sichuan	0.3725	24	0.7438	0.7493	0.6914	0.6702	0.7137	24	0.4664	0.4518	0.4912	0.5289	0.4846	21
Tianjin	1	1	1	1	1	1	1	1	1	1	1	1	1	1
Xinjiang	1	1	1	1	1	1	1	1	1	1	1	1	1	1
Yunnan	0.3529	29	0.5566	0.5638	0.5317	0.5363	0.5471	28	0.3704	0.3610	0.3601	0.3493	0.3602	28
Zhejiang	0.6885	14	0.9178	0.9721	0.9122	1	0.9505	15	0.7631	0.7602	0.7910	0.7474	0.7654	14
Chongqing	0.4930	21	0.6799	0.8230	0.8572	0.9092	0.8174	21	0.4913	0.4520	0.4008	0.4591	0.4508	23

**Table A2:** Undesirable output efficiency in 2013–2016.

DMU	S0_2_				NO_X_				PM2.5			
	2013	2014	2015	2016	2013	2014	2015	2016	2013	2014	2015	2016
Anhui	0.9297	0.8740	0.7760	1	0.6058	0.5719	0.5312	0.6265	1	0.9490	0.8902	0.8773
Beijing	1	1	1	1	1	1	1	1	1	1	1	1
Fujian	1	1	1	1	1	1	1	1	1	1	1	1
Gansu	0.3382	0.1591	0.1532	0.2386	0.5566	0.2954	0.2883	0.3461	0.6201	0.7126	0.6721	0.5998
Guangdong	1	1	1	1	1	1	1	1	1	1	1	1
Guangxi	0.4523	0.7510	1	1	0.5405	0.8242	1	1	0.5177	0.7460	1	1
Guizhou	0.2590	0.1664	0.1690	0.1838	0.5531	0.3646	0.3810	0.3254	0.7726	0.8665	0.9025	0.8204
Hainan	1	1	1	1	1	1	1	1	1	1	1	1
Hebei	0.4742	0.5080	0.4733	0.5393	0.4123	0.4182	0.3909	0.3868	0.7183	0.6896	0.7571	1
Henan	0.5997	0.6273	0.5711	1	0.5212	0.5455	0.5195	0.6470	0.8931	0.9035	0.9310	0.7780
Heilongjiang	0.8025	0.5546	0.5544	0.4472	0.5892	0.3952	0.4230	0.3176	1	0.9886	1	1
Hubei	0.9141	0.9276	0.8321	1	1	1	0.9191	1	0.9130	0.9639	0.9219	1
Huna	0.7580	1	0.8439	0.5692	0.9066	1	1	0.6199	0.6841	1	0.8401	0.7459
Jilin	1	0.8602	0.9496	0.6616	1	0.6288	0.9302	0.5493	1	1	0.9931	1
Jiangsu	1	1	1	1	1	1	1	1	1	1	1	1
Jiangxi	0.4667	0.4741	0.4322	0.6675	0.5543	0.5433	0.5223	0.4672	0.8123	0.8567	0.8078	0.8376
Liaoning	1	1	1	0.5952	1	1	1	0.6376	1	1	1	1
Inner Mongolia	1	1	1	1	1	1	1	1	1	1	1	1
Ningxia	1	1	1	1	1	1	1	1	1	1	1	1
Qinghai	1	1	1	1	1	1	1	1	1	1	1	1
Shandong	0.7511	0.6456	0.7577	0.4510	0.7884	0.7030	0.7710	0.5193	0.9484	0.9269	0.7808	1
Shanxi	0.3145	0.2968	0.3043	0.3705	0.3828	0.3561	0.3801	0.3348	0.9938	0.9488	0.8165	0.7895
Shaanxi	1	1	1	1	1	1	1	1	1	1	1	1
Shanghai	1	1	1	1	1	1	1	1	1	1	1	1
Sichuan	0.6810	0.6860	0.6658	0.6410	1	1	0.9037	0.8798	0.9332	0.8353	1	1
Tianjin	1	1	1	1	1	1	1	1	1	1	1	1
Xinjiang	1	1	1	1	1	1	1	1	1	1	1	1
Yunnan	0.2366	0.1978	0.1925	0.1722	0.4082	0.3443	0.3280	0.2645	1	1	1	1
Zhejiang	0.9751	0.9814	0.8673	1	1	1	1	1	1	1	1	1
Chongqing	0.5752	0.5235	0.4053	0.5404	1	0.8859	0.7284	0.7729	0.7001	0.6376	0.6044	0.6429
Average	0.7843	0.7745	0.7649	0.7693	0.8273	0.7959	0.8006	0.7565	0.9169	0.9342	0.9306	0.9364

## Appendix C

**Table A3:** The four-year average output value of the output indicator.

DMU	GDP	SO2	NOX	PM2.5
Anhui	3763	67,582	137,758	82
Beijing	6888	170,637	246,392	78
Fujian	13,135	1,093,104	1,410,596	88
Gansu	4955	1,067,685	957,801	56
Guangdong	7705	1,131,954	1,105,052	42
Guangxi	10,762	874,526	825,183	55
Guizhou	6167	326,176	478,037	54
Hainan	4394	438,974	666,666	44
Hebei	7305	162,272	295,020	53
Henan	27,730	812,899	1,142,140	62
Heilongjiang	17,120	493,397	607,231	48
Hubei	9419	438,989	724,899	57
Huna	10,600	311,025	372,716	28
Jilin	6860	474,268	505,621	46
Jiangsu	25,776	1,473,850	1,474,476	81
Jiangxi	15,903	1,002,510	1,264,578	78
Liaoning	11,300	505,018	524,611	67
Inner Mongolia	10,758	551,812	514,625	58
Ningxia	29,737	631,019	1,041,478	38
Qinghai	6211	390,209	405,760	50
Shandong	489	28,559	86,693	21
Shanxi	5387	464,693	313,875	62
Shaanxi	11,373	704,740	546,652	64
Shanghai	3215	853,060	461,348	44
Sichuan	3851	602,431	479,713	29
Tianjin	7611	660,034	618,080	70
Xinjiang	1989	495,057	376,648	48
Yunnan	916	143,857	119,698	56
Zhejiang	985	340,306	351,737	48
Chongqing	2881	735,357	771,496	66
